# Revealing Calcium Signaling Pathway as Novel Mechanism of Danhong Injection for Treating Acute Myocardial Infarction by Systems Pharmacology and Experiment Validation

**DOI:** 10.3389/fphar.2022.839936

**Published:** 2022-02-23

**Authors:** Siyu Guo, Yingying Tan, Zhihong Huang, Yikui Li, Weiyu Liu, Xiaotian Fan, Jingyuan Zhang, Antony Stalin, Changgeng Fu, Zhishan Wu, Penglong Wang, Wei Zhou, Xinkui Liu, Chao Wu, Shanshan Jia, Jinyan Zhang, Xiaoxia Duan, Jiarui Wu

**Affiliations:** ^1^ School of Chinese Materia Medica, Beijing University of Chinese Medicine, Beijing, China; ^2^ Xiyuan Hospital, China Academy of Chinese Medical Sciences, Beijing, China; ^3^ Institute of Fundamental and Frontier Sciences, University of Electronic Science and Technology of China, Chengdu, China; ^4^ China-Japan Friendship Hospital, Beijing, China; ^5^ Beijing Zest Bridge Medical Technology Inc., Beijing, China

**Keywords:** Danhong injection, acute myocardial infarction, systems pharmacology, molecular docking, experiment validation

## Abstract

**Introduction:** Danhong injection (DHI) is a traditional Chinese medicine preparation commonly used in the clinical treatment of acute myocardial infarction (AMI). In this study, the active components of DHI and its mechanism in the treatment of AMI were investigated.

**Methods:** The chemical components of DHI were detected by the ultra-high-performance liquid chromatography-linear trap quadrupole-orbitrap-tandem mass spectrometry (UHPLC-LTQ-Orbitrap-MS/MS), and the targets and pathways of DHI in the treatment of AMI were analyzed by systems pharmacology, which was verified by molecular docking and animal experiments.

**Results:** A total of 12 active components of DHI were obtained, and 158 common targets of component and disease were identified by systems pharmacology. Kyoto Encyclopedia of Genes and Genomes (KEGG) analysis results showed that DHI is closely related to the calcium signaling pathway in the treatment of AMI. Molecular docking showed that the key target protein has good binding affinity to related compounds. The experimental results showed that compared with the model group, LVAWs, EF, and FS significantly (*p* < 0.05) increased in the DHI group. The percentage of myocardial infarction significantly (*p* < 0.01) decreased, both in the ventricular and total cardiac regions, and the pathological damage of myocardial tissue also decreased. In addition, the expression of the protein CaMK II decreased (*p* < 0.01) and the expression of SERCA significantly increased (*p* < 0.01).

**Conclusion:** This study revealed that ferulic acid, caffeic acid and rosmarinic acid could inhibit AMI by regulating PLB, CaMK II, SERCA, etc. And mechanistically, calcium signaling pathway was critically involved. Combination of systems pharmacology prediction with experimental validation may provide a scientific basis for in-depth clinical investigation of the material basis of DHI.

## Introduction

Acute Myocardial Infarction (AMI) is an acute and critical disease of the cardiovascular system and is characterized by high morbidity, rapid disease progression, and high mortality, which poses a serious threat to human health (Heron and Anderson, 2016; Khodayari et al., 2019; Wu Wanda. Y et al., 2020). The occurrence of the disease is mostly related to risk factors such as overwork, long-term smoking, heavy alcohol consumption, hypertension and hyperlipidemia (van der Schoot et al., 2020). Pathophysiological studies have shown that rupture of coronary atherosclerotic plaques, interruption of epicardial coronary blood flow, and occlusive thrombosis can cause AMI, leading to myocardial ischemia, myocardial cell death, and impaired cardiac function (Davies, 2000; Weil and Neelamegham, 2019). Although the application of thrombolysis, percutaneous coronary intervention (PCI), and integrated treatment with traditional Chinese and Western medicine has significantly improved the survival rate of AMI patients, there are still some reasons that impair the revascularization of AMI and lead to irreversible death of myocardial tissues and cells, which seriously affects the prognosis of AMI patients (Do et al., 2015; Magnuson et al., 2017). Recent studies had shown that sodium-calcium exchange plays an important role in Ca^2+^ efflux mechanism of ventricular cardiomyocytes (Santana et al., 2016). In addition, intracellular Ca^2+^ overload occurred during the period of myocardial ischemia (Tsai et al., 2020). Moreover, studies have shown that the calcium signaling pathway is closely related to acute myocardial infarction (Guo et al., 2021a).

In Chinese medicine, AMI belongs to “angina pectoris” and “chest pain.” With the development of traditional Chinese medicine, the prevention and treatment of AMI by traditional Chinese medicine has attracted more and more attention (Liu et al., 2011; Spatz et al., 2018; Lu et al., 2019). DHI is a kind of Chinese patent medicine extracted from two Chinese medicines, *Salvia miltiorrhiza Bunge [Lamiaceae; Salviae miltiorrhizae radix et rhizoma]* (Danshen) and *Carthamus tinctorius L. [Asteraceae; Carthami Flos]* (Honghua), by modern technology, and is widely used in the treatment of various diseases such as AMI (You et al., 2019; Zhang Chi et al., 2020). DHI mainly contains chemical components such as tanshinone, danshensu, safflower yellow, and salvianolic acid, which can promote blood circulation, counteract platelet aggregation, improve the hemodynamic status, alleviate myocardial ischemia, and reduce atherosclerosis (Feng et al., 2019). DHI could positively and effectively improve the clinical symptoms of AMI patients (Liao et al., 2015). In addition, recent study shown that DHI could improve myocardial injury in patients with PCI perioperative period (Xing et al., 2021). Furthermore, a systematic review concluded that in the PCI postoperative, 30 ml dose of DHI could inhibit interleukin-6 (IL-6) levels, a course of 14 days was most effective than other treatment courses for AMI patients with high hypersensitive C-reactive protein (hs-CRP), and 10-day postoperative treatment period produced better curative effect for AMI patients with obvious abnormalities of creatine kinase (CK)-MB (He et al., 2021). However, there is no in-depth report on the mechanism of DHI in the treatment of AMI.

**FIGURE 1:**
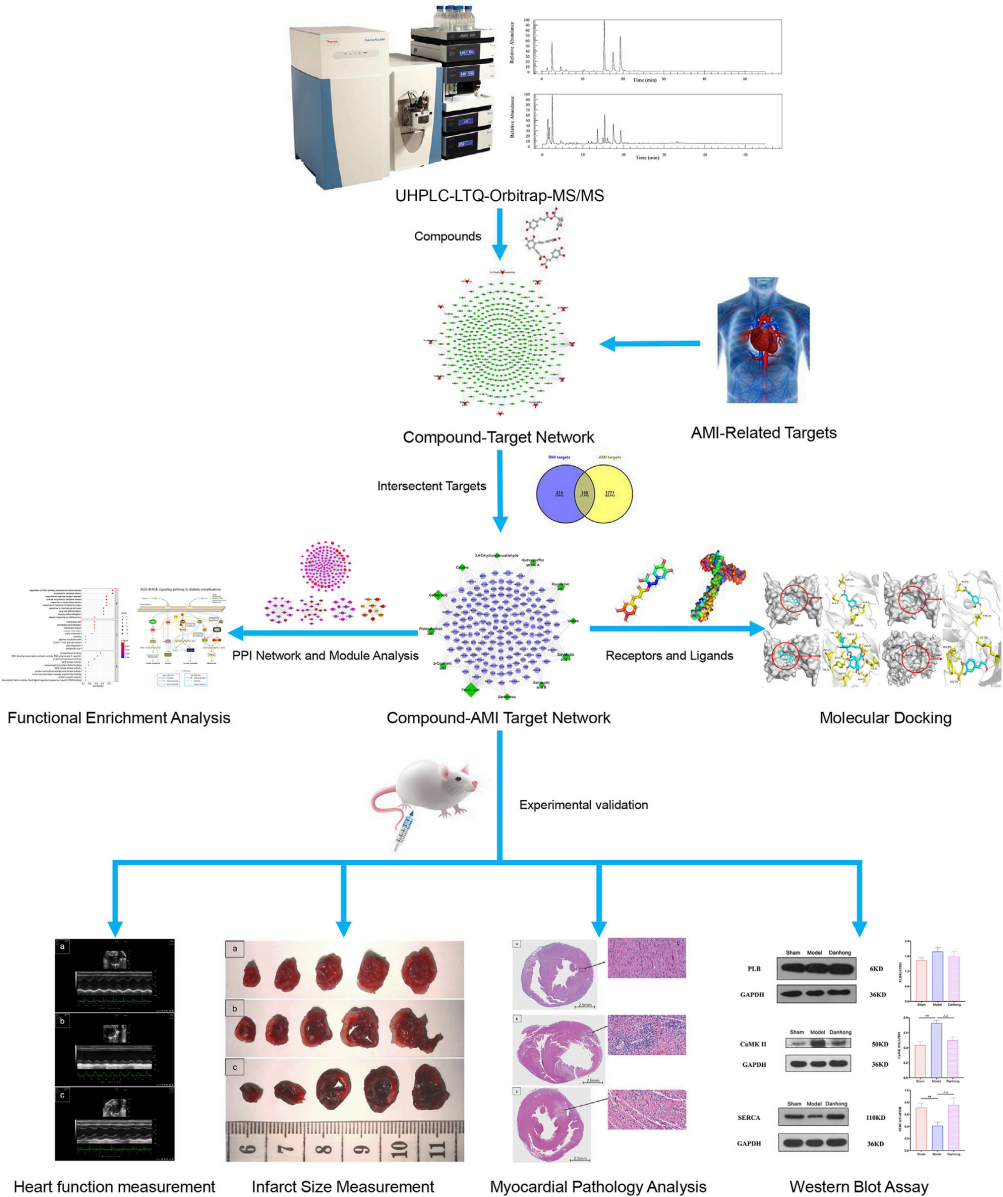
Workflow diagram of this study.

In recent years, systems pharmacology has provided the possibility of a better understanding the complex biological characteristics of Chinese medicine in the treatment of various diseases (Gao et al., 2020). Systems pharmacology mainly relies on big data analysis to collect information about the active ingredients, targets and diseases to construct the compound-target network, disease-target network and compound-target-disease network to elucidate the mechanism of traditional Chinese medicine in the treatment of diseases at molecular level. Furthermore, these networks are analyzed based on their topological characteristics, and the key targets corresponding to the components and the related biological processes are determined as a whole (Guo et al., 2020). In addition, systems pharmacology can explain the “multi-component, multi-target and multi-pathway” mechanism of traditional Chinese medicine prescriptions at the systems level, which is consistent with the “holistic concept” of traditional Chinese medicine (Zhou et al., 2018; Zhang et al., 2019; Niu et al., 2021). Wu et al., found that Gegen (*Pueraria montana var. lobata (Willd.) Maesen ＆ S.M.Almeida ex Sanjappa ＆ Predeep [Fabaceae; Puerariae Lobatae Radix]*) can be used to treat hypertension by influencing the proliferation module, apoptosis module, and inflammation module through the systems pharmacology method (Wu et al., 2020). In this study, the combination of liquid chromatography-mass spectrometry, systems pharmacological analysis, and experimental validation was used to explore the important role of the calcium signaling pathway in AMI, revealing the mechanism of drugs on diseases more comprehensively, and provide new ideas for the in-depth study of AMI. On this basis, the workflow diagram of this study was shown in Figure 1.

## Materials and Methods

### Screening of DHI Componets

In this study, UHPLC-LTQ-Orbitrap-MS/MS method was used to identify the components of DHI. The reagents and instruments used were as follows: DHI, provided by Shandong Danhong Pharmaceutical Co., LTD., specification 10 ml/piece. Ultimate 3000 ultra performance liquid chromatography and LTQ Orbitrap XL mass spectrometer (equipped with an electrospray ion source (ESI) and xcalibur 2.1 Chemstation) were purchased from Thermo Scientific, United States. Mass spectrometric conditions: positive and negative ion detection mode, electrospray ion source (ESI), sheath gas and auxiliary gas were nitrogen gas with purity of >99%; Sheath gas flow rate 40 U, auxiliary gas flow rate 20 U; Collision gas was helium with purity of >99.99%; Ionization source voltage, 3.0 kV; Ion source temperature: 350°C; Drying gas flow rate 15 L·min-1; Collision voltage: 6–10 V; First order mass spectra were obtained by Fourier transform with high-resolution and full scan (FT) with a mass ranging from M/Z: 50–1200; Detection resolution: 3,000; Liquid phase conditions were: flow rate: 0.200 ml/min; Needle wash: methanol; Column oven temperature: 30°C; Autosampler injection volume: 4.00 μl; Mobile phase conditions were 0.1% formic acid (a), acetonitrile (b): 0.01–5.00 min B: 15–20, 5.00–20.00 min B: 20–35, 20–40 min B: 35–55, 40–45 min B: 55–35, 45–55 min B: 55–15. The column was: waters HSS T3 UPLC C18 column (1.7 µm, 2.1 × 100 mm).

In addition, PubMed (https://pubmed.ncbi.nlm.nih.gov/) and CNKI (https://www.cnki.net/) databases were used to screen the main compounds of DHI. Then, PubChem (https://pubchem.ncbi.nlm.nih.gov/) database was used to find the SMILES structural information of candidate compounds (Kim et al., 2016). At the same time, ChemDraw (http://www.chemdraw.com.cn/) software was used to draw the 2D structure of the candidate compounds (Evans, 2014).

### Screening of DHI Compounds Targets

In this study, the following three databases were mainly used to find known or predicted targets related to compounds: SuperPred (http://prediction.charite.de/) (Nickel et al., 2014), SwissTargetPrediction (http://www.swisstargetprediction.ch/) (Gfeller et al., 2014) and BATMAN-TCM (http://bionet.ncpsb.org/batman-tcm/) (Liu et al., 2016). In addition, the UniProt (http://www.uniprot.org/) online database was used to convert the obtained protein names into gene names, and the source species was restricted to “Homo sapiens,” so that the names were standardized for later processing of the data (Liu et al., 2019).

### Screening of AMI Targets

The human gene data associated with AMI are from the following six databases: DigSee database (http://210.107.182.61/geneSearch/) (Kim et al., 2017), DisGeNet database (http://www.disgenet.org/search) (Piñero et al., 2017), OMIM database (https://omim.org/) (Amberger et al., 2015), TTD database (https://db.idrblab.org/ttd/) (Chen, 2002), MalaCards database (https://www.malacards.org/) (Rappaport et al., 2017), and GEO database (https://www.ncbi.nlm.nih.gov/gds/) (Barrett et al., 2012). The duplicate values of human gene targets related to AMI obtained from the above six databases were deleted for further analysis.

### Protein-Protein Interaction Analysis

In this study, the AMI targets and DHI compound targets were crossed and the same targets were uploaded to the STRING 11.0 database (Szklarczyk et al., 2017). In addition, the species source was “Homo sapiens,” and the confidence score was set to be greater than 0.7.

### Network Construction and Module Analysis

In this study, Cytoscape 3.7.1 (https://cytoscape.org/) was used to construct the following six visualization networks: *1*) compound-target network; *2*) compound-AMI target network; *3*) PPI network of the common target of DHI compound and AMI, namely DHI-AMI target protein-protein interaction network; *4*) modular analysis network; *5*) DHI-key compound-key target-pathway network. In addition, the NetworkAnalyzer tool in Cytoscape was used to perform a topological analysis of the network, including “degree,” “betweenness” and “closeness.” The higher the value of these parameters, the more important the node is in the network. Moreover, on this study, the MCODE plug-in in Cytoscape software was used to analyze the PPI network (Chen et al., 2021). The default parameters are: Degree Cutoff = 2; Node Score Cutoff = 0.2; K-Core = 2; Max. Depth = 100.

### Functional Enrichment Analysis

To better understand the biological functions and signal transduction pathways of the potential core targets of DHI in the treatment of AMI, the “Bioconductor package” of R 3.6.1 software and Kyoto encyclopedia of genes and genomes (KEGG) was used for the for Gene Ontology (GO) and pathway enrichment analysis, respectively (Robinson et al., 2009).

### Molecular Docking

The PubChem database platform was used to download the main compound files in SDF format, and the ChemDraw software converted the SDF format to mol2 format. Afterward, Autodock Tools 1.5.6 software was used to load the processed mol2 format files, set up rotatable bonds, etc., and finally, save them as PDBQT format files (Morris et al., 2009). This study screened the protein conformations of key targets from RCSB PDB (https://www.rcsb.org/) (Goodsell et al., 2020). The screening conditions are: *1*) the protein structure obtained by the X-ray crystal diffraction method; *2*) the crystal resolution of the protein is less than 3Å; *3*) the biological source of the protein should be human; *4*) preferentially select the protein structure reported in the literature on molecular docking. At the same time, Notepad++ (https://notepad-plus-plus.org/) and AutoDockTools were used to remove water molecules and co-crystallized original ligand molecules, adding hydrogen and charge, merge non-polar hydrogen, and determine the location of active pockets. Then, Autodock Vina 1.1.2 was used to perform molecular docking calculations with the processed ligands and receptors (Trott and Olson, 2009). Finally, PyMol 2.3.2 (https://pymol.org/2/) was used to visualize the molecular docking results (Yuan et al., 2016).

### Reagents and Instruments

DHI, provided by Shandong Danhong Pharmaceutical Co., LTD., specification 10ml/piece. Nitrotetrazolium chloride blue (N-BT), Amresco Co, Lot 2541C012. Tp-213 electronic balance, Beijing Sartorius Instrument System Co., LTD. Dt-2000 electronic balance, American Shuangjie Brothers Co., LTD. Mpids-500 Color Pathological Image Analysis System, Beijing Air Sea Company; Canon EOS 5D Mark ⅲ, Canon China Co., LTD. Vevo 3100 High resolution Ultrasound Imaging System for Small Animals, Visual Sonics Inc.

### Modeling and Drug Administration

A total of 80 male SD rats weighing 180–200 g were purchased from Scxk (Beijing) Biotechnology Co., Ltd. (animal qualification certificate no.: SCXK (Beijing) 2019-0010). Rats were maintained on a 12 h light/dark cycle, at 22 ± 2°C and controlled humidity. Besides, they had *ad libitum* access to water and rodent food. Animal care, surgery, and handling procedures were performed in accordance with the regulations of the Ministry of Science and Technology of the People’s Republic of China ([2006] 398) and were approved by the Animal Care Committee of Xiyuan Hospital of the China Academy of Chinese Medical Sciences (SYXK [JING] 2019-0010).

Rats were anesthetized by intraperitoneal injection of 12 ml/kg 2% sodium pentobarbital and fixed in the supine position on a plate in. Then, a thoracotomy was performed at the fourth and fifth intercostal points on the left side of the rat, and the pericardium was opened. The thoracic cavity was gently pressed to extrude the heart, and the left anterior descending coronary artery was ligated with a 0 suture approximately 2 mm below the left atrial appendage. The heart was immediately returned to the thoracic cavity, and the opened skin was sutured. At the same time, rats in the sham group only thread and did not ligate.

After successful modeling, rats were randomly assigned to different groups such as sham (*n* = 20), model (*n* = 30), and DHI 6 ml/kg (*n* = 30). Rats in each group received a continuous intraperitoneal injection for seven consecutive days, and the sham group and the model group received normal saline in the same manner.

### Heart Function Measurement

A small animal ultrasound instrument was used for cardiac ultrasonography, and echocardiography was performed 3 h after the last administration. After anesthesia, rats were fixed in the supine position on a thermostatic heating plate, and their limbs were connected to electrocardiogram electrodes for monitoring heart rate and recording the electrocardiogram. Cardiac ultrasound was performed using a Vevo 3100 small animal ultrasound instrument. After the chest hair was removed, the body of the rats was tilted 30° to the left and an ultrasound coupling agent was applied. The ultrasonic probe is placed on the left side of the sternum at an angle of 10°–30° to the midline of the sternum, showing the long axis section of the left ventricle of the sternum. Apply M-mode ultrasound to the long axis view of the left ventricle, run the sampling line through the left anterior and posterior sidewall at the level of the chordal chordae of the mitral valve, record and measure the motion curve of the left ventricle. Then the probe is rotated 90° clockwise, making moderate adjustments to show that the left ventricle is short. Using anatomical M-mode ultrasound on the short-axis section of the left ventricular papillary muscle, the sampling line passes through the anterior septum and posterior wall. Guided by two-dimensional images, the m-shaped curve of left ventricular motion was recorded and measured, including left ventricular ejection fraction (EF), left ventricular short axis shortening rate (FS%), and left ventricular anterior wall thickness (LVAWd and LVAWs) in the end diastolic and end systolic stages.

### Infarct Size Measurement

Three hours after the last administration to the rats of each group, the heart was immediately removed, the residual blood in the cardiac cavity was flushed with normal saline, the excess fluid was absorbed with filter paper, and then weighed. The ventricle was cut evenly from the sulcus parallel to the coronary artery under the ligation line into five equal thickness slices. Then, these slices were weighed separately, placed in 0.2% nitrotetrazolium blue dye solution (N-BT), and stained for 2–3 min in the dark at room temperature. Subsequently, the camera was used to photograph the anterior and posterior sides of five myocardiums to obtain images, and the pathological color image analysis system was used to measure each piece on both sides of the myocardial infarct size (N-BT non-stained area) and non-infarct size (N-BT stained area). At the same time, total ventricular muscle size, total infarct size, and percentage of infarct size to ventricular size and whole heart size were also calculated.

### Myocardial Pathology Analysis

Five rats from each group were selected for cardiac histopathological observation. The method was as follows: after anesthesia, the hearts of the rats were removed, the right ventricle was stripped and discarded, and the myocardial tissue in the infarct junction area was collected. Cardiac tissue specimens were placed in a prepared 4% formaldehyde fixative. After dehydration, they were conventionally embedded in paraffin and sectioned. Then the tissues were stained with HE and Masson. The degree of changes in the myocardium was observed under the microscope.

### Western Blot Assay

RIPA lysate (Thermo Scientific) was used to extract protein samples, and protein concentration (Pierce) was determined by the BCA method. The SDS-PAGE gel was prepared using the Mini-PROTEAN Tetra hand filling system (Bio-rad), and each well was loaded with 30 μg normalized final loading concentration. After electrophoresis, the protein was transferred to the PVDF membrane (Millipore), and the corresponding antibody was incubated to achieve immunoaffinity between antibody and antigen. Developing with ECL (Thermo Scientific), and exposing with Kodak Carestream to obtain protein imprinted bands.

### Statistical Analysis

Data are expressed as “mean ± standard deviation ( $\bar{x}$ ±s),” and SPSS 25.0 statistical analysis software is used for data analysis and processing. For data conforming to the normal distribution, one-way analysis of variance (ANOVA) is used for two or more groups. T Homogeneity of variance was tested using Student-Newman-Keuls and the uneven variance was tested using Tamhane’s T2; data that did not conform to the normal distribution was tested using a non-parametric test. Differences with *p* < 0.05 were considered statistically significant.

## Results

### Identification of Major Compounds in Compound DHI by UHPLC-LTQ-Orbitrap-MS/MS

**FIGURE 2:**
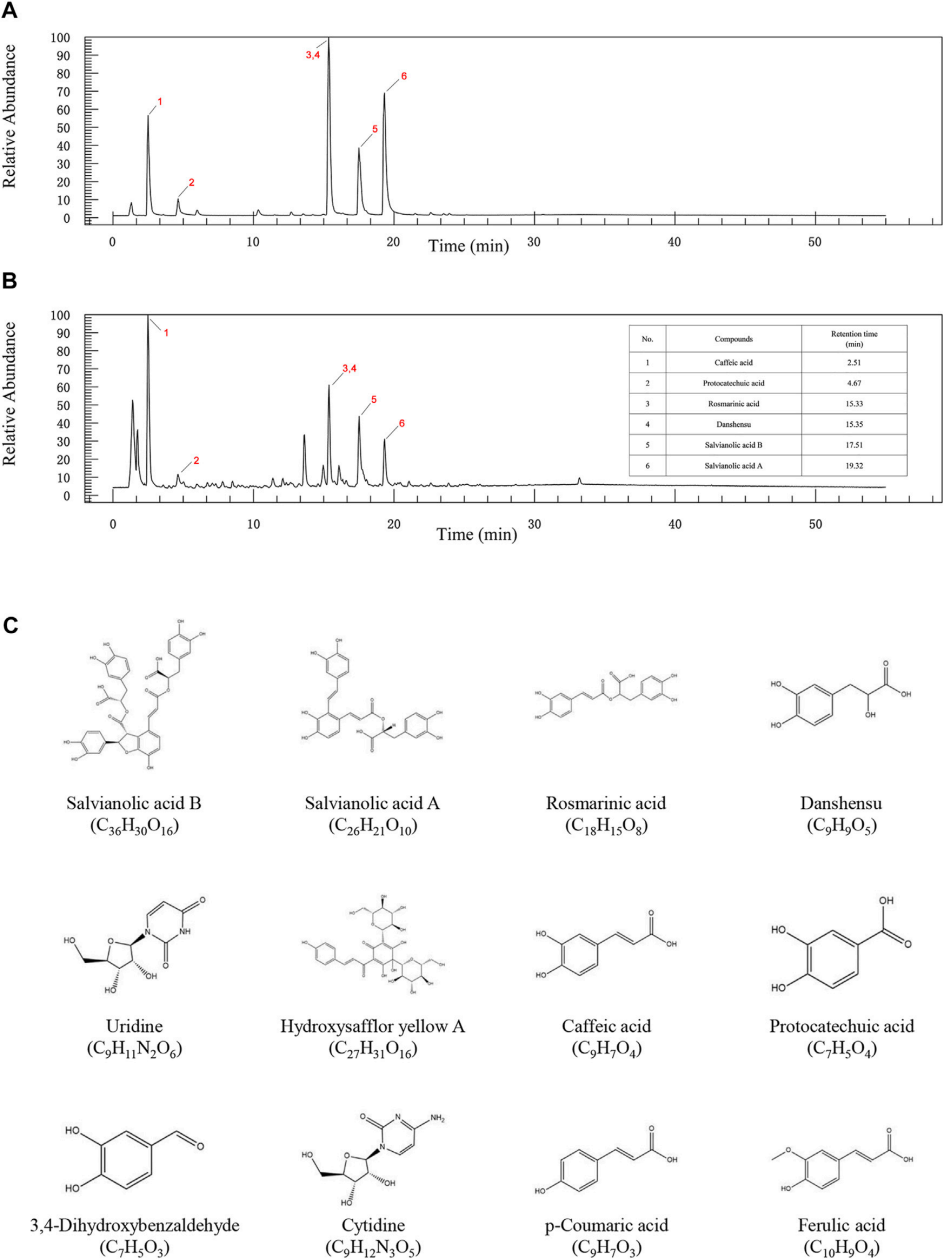
The UPLC-QE-Orbitrap-MS chromatography of standard substances **(A)** and DHI **(B)**. **(C)** 2D structure diagram of key compounds.

After the compound screening, a total of 12 major compounds were incorporated into this study (Zhao et al., 2017; Li et al., 2019; Xu et al., 2019), including Salvianolic acid A, Salvianolic acid B, Danshensu, Protocatechuic acid, Rosmarinic acid, and Caffeic acid, which were also detected in UHPLC-LTQ-Orbitrap-MS/MS analysis (Figure 2 and Supplementary Tables S1, S2).

### Compound-Target Network of DHI and Compound-AMI Target Network

**FIGURE 3:**
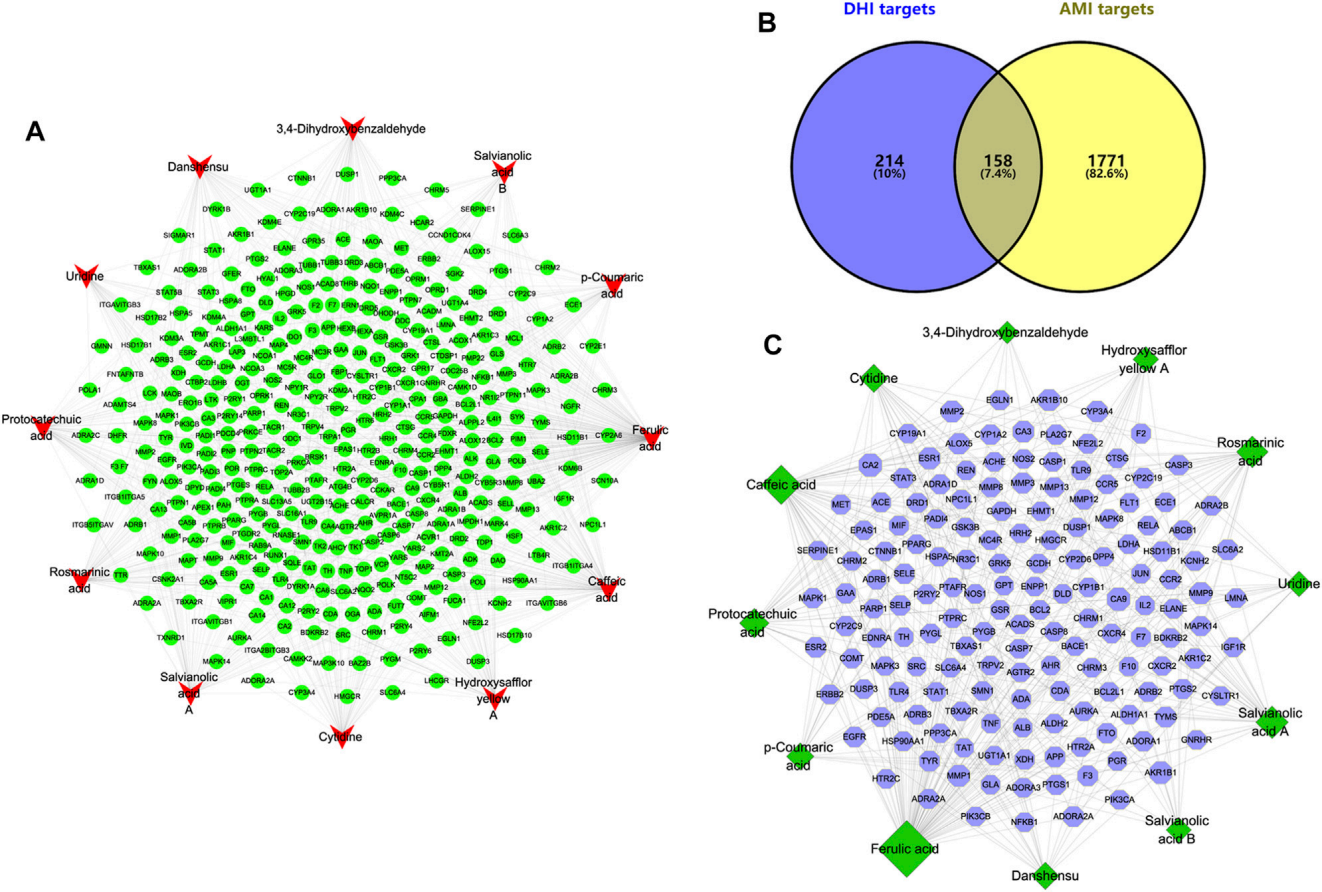
Compound-target network of DHI and Compound-AMI target network. **(A)** Compound-target network of DHI. The red represents the main compound of DHI, and the green represents the corresponding target of the compound. **(B)** Venn diagram of DHI targets and AMI targets. **(C)** Compound-AMI target network. The green represents the main compound of DHI, and the blue represents the common target of compound and AMI.

By searching the SuperPred, SwissTargetPrediction, and BATMAN-TCM databases, a total of 372 targets were found to be associated with the compound. The compound-target network of DHI is shown in Figure 3A. It consists of 384 nodes (12 compound nodes, 372 target nodes) and 708 edges. This network shows that a single target can be regulated by multiple compounds, which could play a vital role in the treatment of AMI. Indeed, a single compound may act on multiple potential targets.

In this study, mainly AMI-related targets were retrieved from the five databases DigSee, DisGeNet, OMIM, TTD, and MalaCards, and the AMI differential genes of published articles were combined and then duplicate data were detected (Guo et al., 2021b). A total of 1929 potential targets related to AMI were obtained. At the same time, the obtained AMI potential targets and compound targets were intersected and uploaded to Cytoscape to construct a compound-AMI target network (Figure 3B). As shown in Figure 3C, this network contains 170 nodes (12 compound nodes, 158 target nodes) and 318 edges. In addition, the topological characteristics of the network were analyzed. The results show that the top three compounds in terms of “degree,” “betweenness” and “closeness” are ferulic acid, caffeic acid and salvianolic acid A, respectively. And the three largest targets with topological parameters are carbonic anhydrase 9 (CA9), carbonic anhydrase 2 (CA2) and aldo-keto reductase family 1 member B1 (AKR1B1). These compounds and targets may play an important role in the treatment of AMI with DHI.

### PPI Network Construction and Module Analysis

**FIGURE 4:**
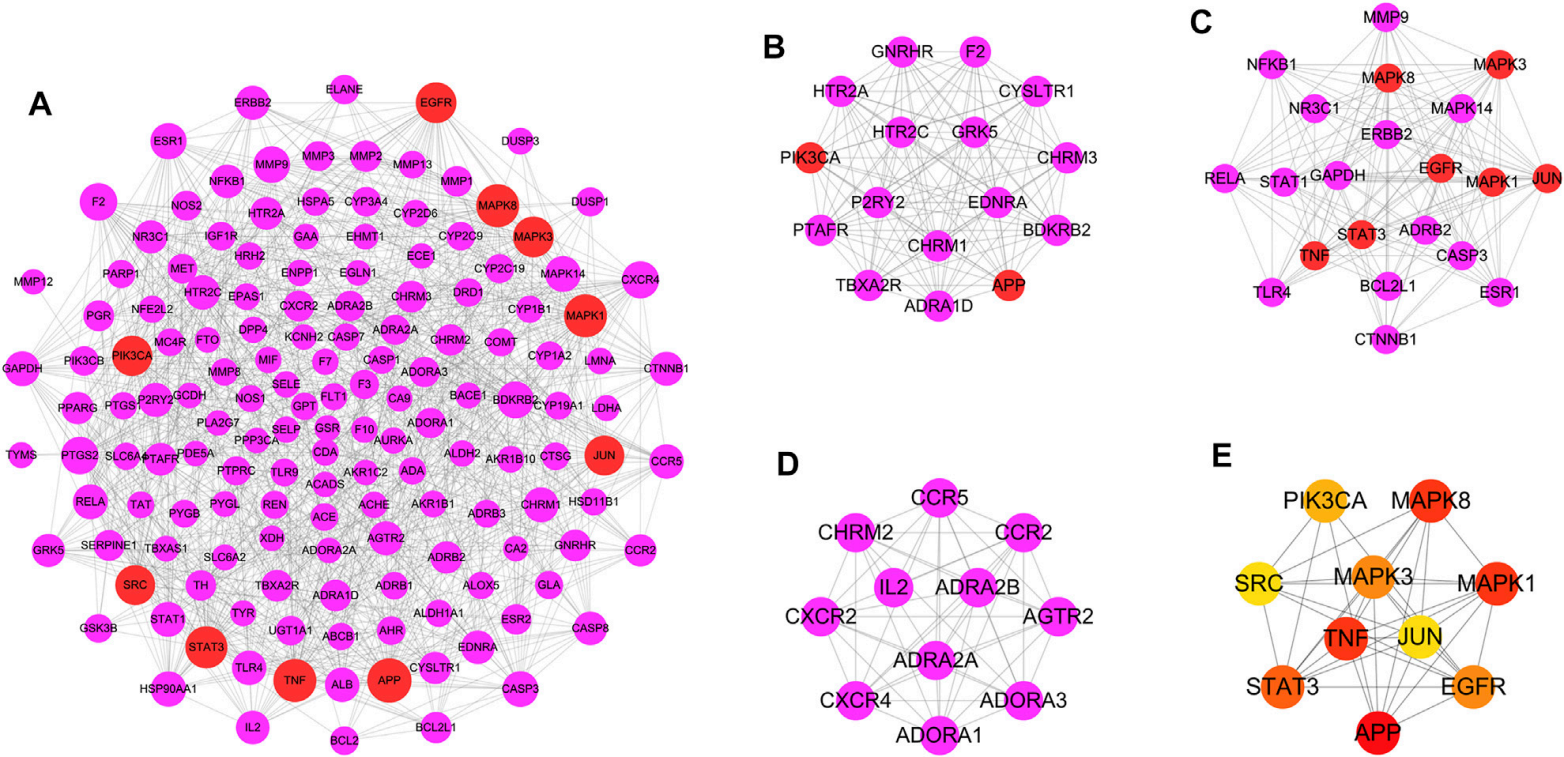
Protein-protein interaction network and module analysis. **(A)** DHI-AMI target protein-protein interaction network. **(B)** Module 1. **(C)** Module 2. **(D)** Module 3. The purple represents the common target of DHI and AMI, and the red represents the top 10 targets. **(E)** The interconnection diagram of 10 key targets. From yellow to red, the darker the color, the higher the MCC score.

In this study, the AMI target and the DHI target were crossed and imported into the STRING database platform to construct a DHI-AMI target protein-protein interaction network to better understand the complex interactions between common targets. As shown in Figure 4A, the network contains 151 nodes and 859 edges; the node’s size is proportional to the degree value. In this study, the top ten nodes with a median network value were selected as important nodes: APP, MAPK1, TNF, MAPK8, STAT3, EGFR, MAPK3, PIK3CA, JUN, SRC (Supplementary Table S3).

Traditional Chinese medicine has the complex characteristics of multi-component and multi-target, and the module analysis based on the “law of similar attraction” is considered one of the most powerful methods to explain the mechanism of Chinese medicine (Nie et al., 2020). A network module means that the connection between the nodes of the sub-network is closer than the connection with the rest of the network, and it has the characteristics of high interconnection, which helps to find the structure and function information of similar proteins in the network (Guimerà and Nunes Amaral, 2005). In this study, a modular analysis of the DHI-AMI target protein-protein interaction network was performed. The first three modules with MCODE scores greater than 9.00 were finally selected as important modules (Figures 4B–D). Ten potential key targets are mainly enriched in the first two modules; namely, PIK3CA and APP in module 1; MAPK1, TNF, MAPK8, STAT3, EGFR, MAPK3, and JUN in module 2. SRC is not enriched in important modules.

### Functional Enrichment Analysis

**FIGURE 5:**
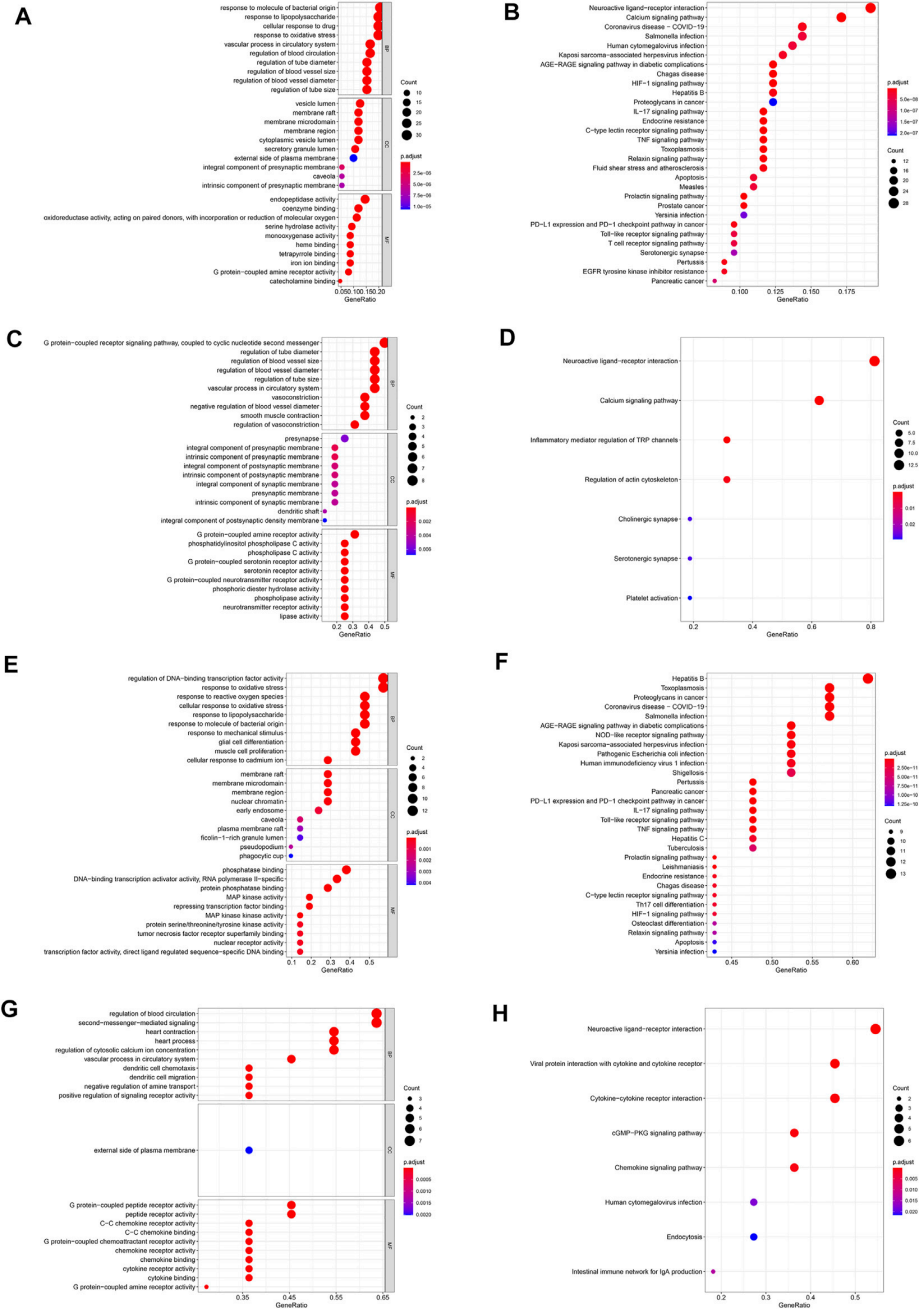
GO and KEGG pathway enrichment analysis. **(A)** Go enrichment analysis of protein-protein interaction targets. **(B)** KEGG pathway enrichment analysis of protein-protein interaction targets. **(C)** Go enrichment analysis of module 1. **(D)** KEGG pathway enrichment analysis of module 1. **(E)** Go enrichment analysis of module 2. **(F)** KEGG pathway enrichment analysis of module 2. **(G)** Go enrichment analysis of module 3. **(H)** KEGG pathway enrichment analysis of module 3.

In this study, GO enrichment and KEGG pathway enrichment analysis were performed for the DHI-AMI target protein-protein interaction network. The three important modules, in order to better understand the cellular component (CC), molecular function (MF), biological process (BP), and signaling pathways associated with the treatment of AMI with DHI. According to the corrected *p*-value (*p* < 0.05), this study only displays the top 10 GO elements in the legend. The results of GO functional enrichment analysis showed that protein-protein interaction targets were mainly related to regulation of blood vessel size, blood circulation, cell membrane, and enzyme activity (Figure 5A); module 1 was mainly related to G protein-coupled receptors, blood vessel diameter, vasoconstriction, intracellular calcium ions, synapses, G protein-coupled amine receptors, and phospholipases (Figure 5C); module 2 was mainly related to oxidative stress response, cell membrane, phosphatase binding, and MAP kinase activity (Figure 5E); module 3 was mainly related to the regulation of blood circulation, dendritic cell chemotaxis, signaling receptor activity, outer plasma membrane, C-C chemokines, and G protein coupling (Figure 5G).

Further, KEGG pathway enrichment analysis was performed for 151 protein-protein interaction targets, and a total of 153 pathways were screened (adjusted *p*-value < 0.05). In this study, only the top 30 pathways are shown in the legends. The results of the KEGG pathway enrichment analysis show (Figure 5B) that the protein interaction targets are mainly enriched in the calcium signaling pathway, IL-17 signaling pathway, TNF signaling pathway, and other signaling pathways. These pathways are mainly related to endocrine system, signal transduction and immune system.

Through the KEGG pathway enrichment analysis of 16 targets in module 1, a total of seven signaling pathways were screened (adjusted *p*-value < 0.05), which are mainly enriched in neuroactive ligand-receptor interaction, calcium signaling pathway, and other signaling pathways. These signaling pathways are mainly related to signal transduction, nervous system and immune system (Figure 5D). Through the KEGG pathway enrichment analysis of 21 targets in module 2 (adjusted *p*-value < 0.05), a total of 141 signaling pathways were screened, which are mainly enriched with toxoplasmosis, AGE-RAGE signaling pathways in diabetic complications, IL-17 signaling pathways and so on. These signaling pathways are mainly related to infectious diseases, cancer, immune system and endocrine system (Figure 5F). Through the KEGG pathway enrichment analysis of 11 targets in module 3 (adjusted *p*-value < 0.05), a total of eight signaling pathways were screened, which are mainly enriched in the interaction of viral proteins with cytokines and cytokine receptors, interaction between cytokine and cytokine receptors and other signaling pathways. These pathways are mainly related to signaling molecules, signal transduction and the immune system (Figure 5H).

### Molecular Docking

**FIGURE 6:**
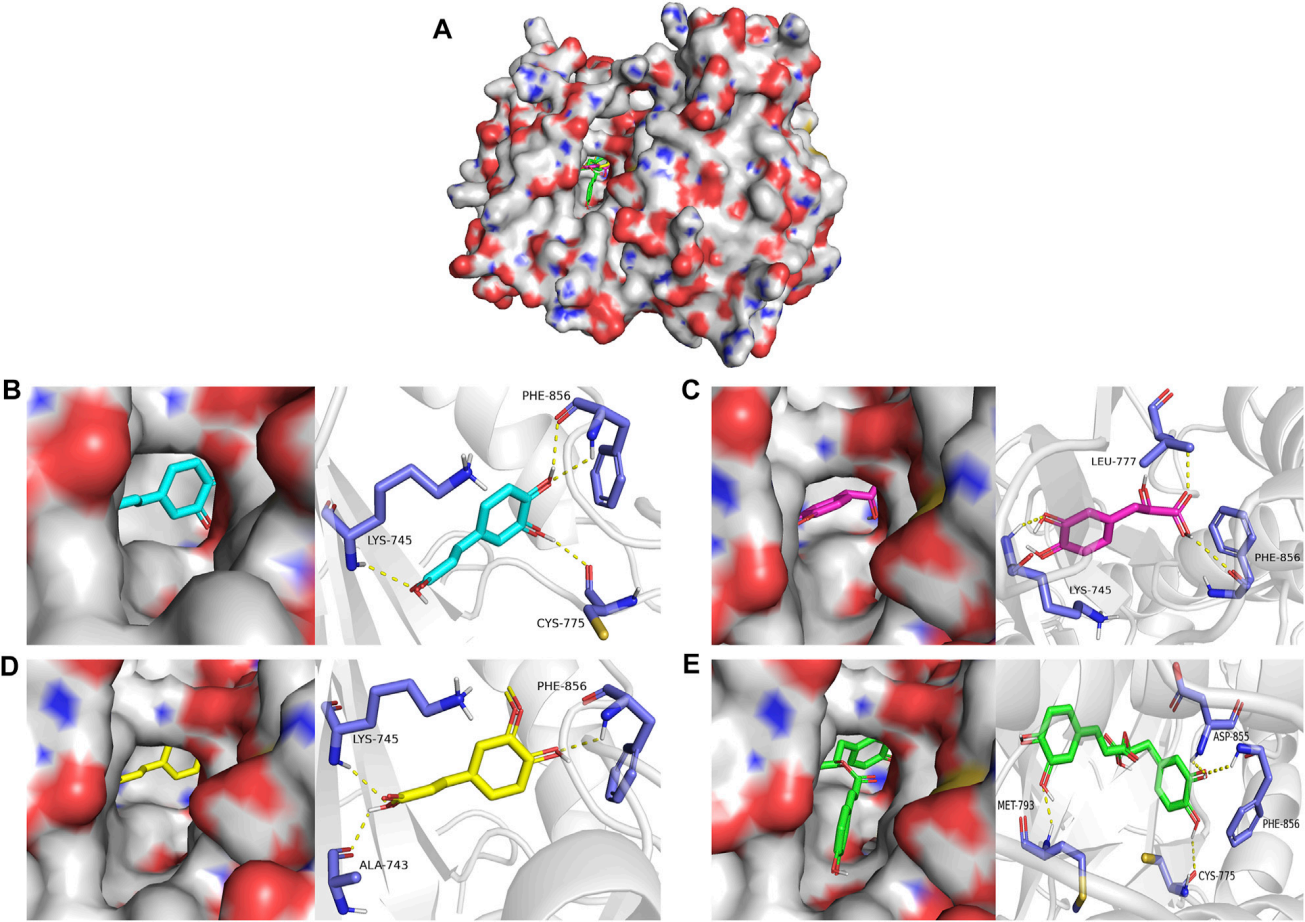
**(A)** Molecular docking of four compounds with EGFR. **(B)** Molecular docking of caffeic acid with EGFR. **(C)** Molecular docking of danshensu with EGFR docking diagram. **(D)** Molecular docking of ferulic acid with EGFR. **(E)** Molecular docking of rosmarinic acid with EGFR.

Molecular docking was used to explore the binding methods of ten key targets and corresponding compounds. The crystal structures of 10 key targets were retrieved and downloaded from PDB database and saved as PDB format file. In addition, the 3D structures of the corresponding compounds (caffeic acid, ferulic acid, rosmarinic acid, salvianolic acid B, danshensu, cytidine, salvianolic acid A) were downloaded from PubChem database. The molecular docking of four compounds with EGFR was analyzed as an example. As shown in Figure 6, caffeic acid forms three hydrogen bonds with residues PHE-856, LYS-745, and CYS-775 on EGFR protein; danshensu forms three hydrogen bonds with residues LEU-777, PHE-856 and LYS-745 on EGFR protein; ferulic acid forms three hydrogen bonds with residues ALA-743, PHE-856 and LYS-745 on EGFR protein; rosmarinic acid forms four hydrogen bonds with MET-793, CYS-775, PHE-856 and ASP-855 residues on the EGFR protein. Interestingly, all four compounds shared some similar binding sites. In addition, the docking scores of all ligand and receptor combinations in this study were greater than −5.520 (Hsin et al., 2013), indicating that the key target protein and corresponding compounds have good binding affinity (Table 1).

**TABLE 1:** Basic information of docking results of 10 key targets with related compounds.

Target	PDB ID	Compound	Affinity (kcal/mol)
APP	1AAP	Caffeic acid	−6.8
		Ferulic acid	−6.0
		Rosmarinic acid	−7.4
		Salvianolic acid B	−8.8
EGFR	3W2S	Caffeic acid	−7.3
		Danshensu	−6.6
		Ferulic acid	−7.0
		Rosmarinic acid	−9.5
JUN	1JNM	Salvianolic acid B	−6.4
MAPK1	5WP1	Caffeic acid	−6.6
		Cytidine	−6.3
		Ferulic acid	−6.6
MAPK3	4QTB	Ferulic acid	−6.9
MAPK8	3PZE	Rosmarinic acid	−7.6
PIK3CA	3HHM	Caffeic acid	−7.0
SRC	1Y57	Salvianolic acid A	−8.1
STAT3	6NJS	Caffeic acid	−5.8
		Ferulic acid	−5.9
		Salvianolic acid A	−7.5
TNF	1TNF	Rosmarinic acid	−9.3

### DHI-Key Compound-Key Target-Pathway Network.

**FIGURE 7:**
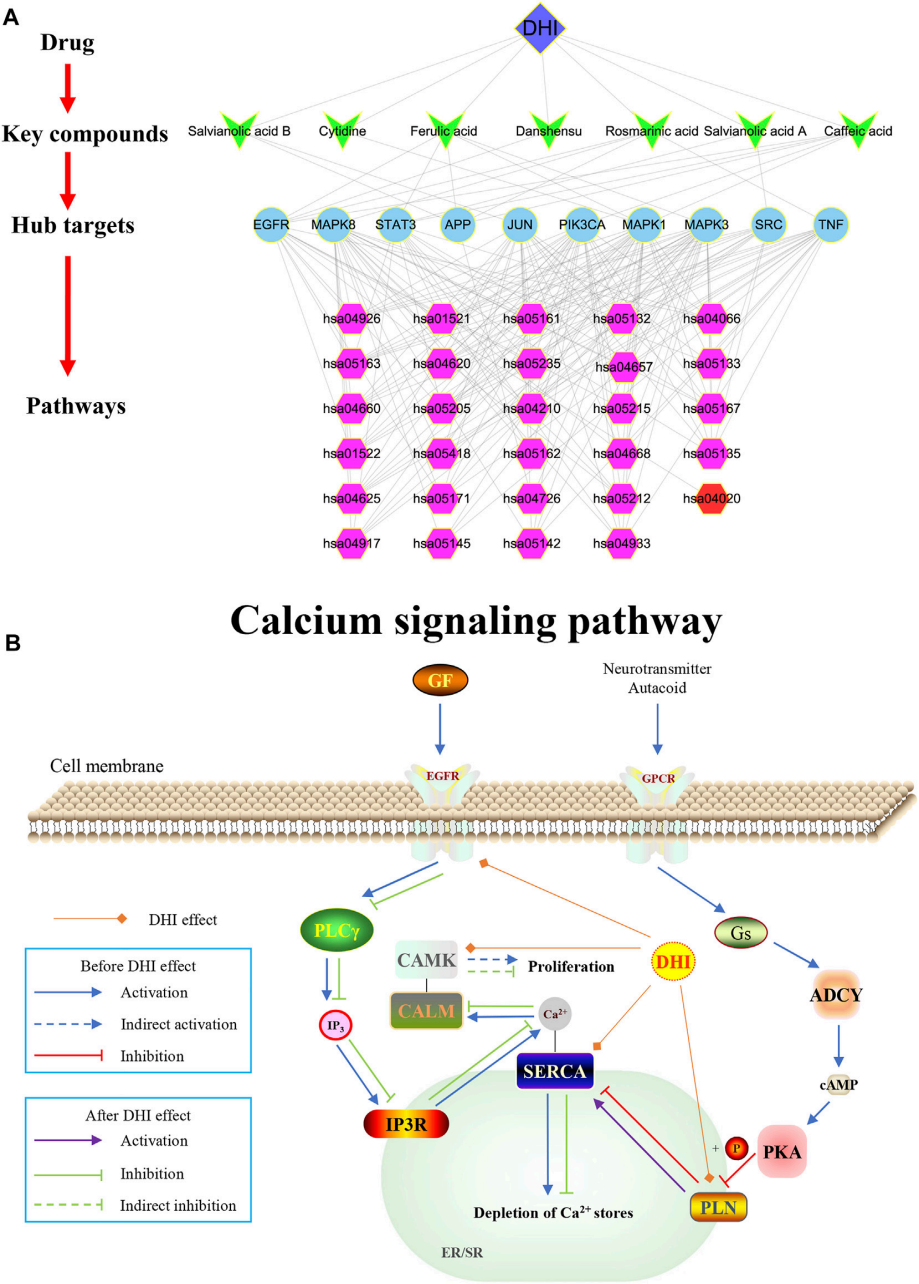
**(A)** DHI-key compound-key target-pathway network. The blue diamond represents DHI; Cyan V-type represents the key compound; the light blue circle represents the key target; the purple hexagon represents the pathways enriched by key targets; the red hexagon represents the calcium signaling pathway. **(B)** Schematic diagram of the calcium signaling pathway (hsa04020).

In this study, Cytoscape software was used to construct a DHI-key compound-key target-pathway network to systematically reveal the mechanism of DHI in the treatment of AMI. As shown in Figure 7A, the DHI-key compound-key target-pathway network includes 47 nodes (one drug node, seven compound nodes, 10 core target nodes, and 29 pathway nodes) and 194 edges. In addition, the calcium signaling pathway was also significantly enriched (Figure 7B).

### Heart Ultrasound

**FIGURE 8:**
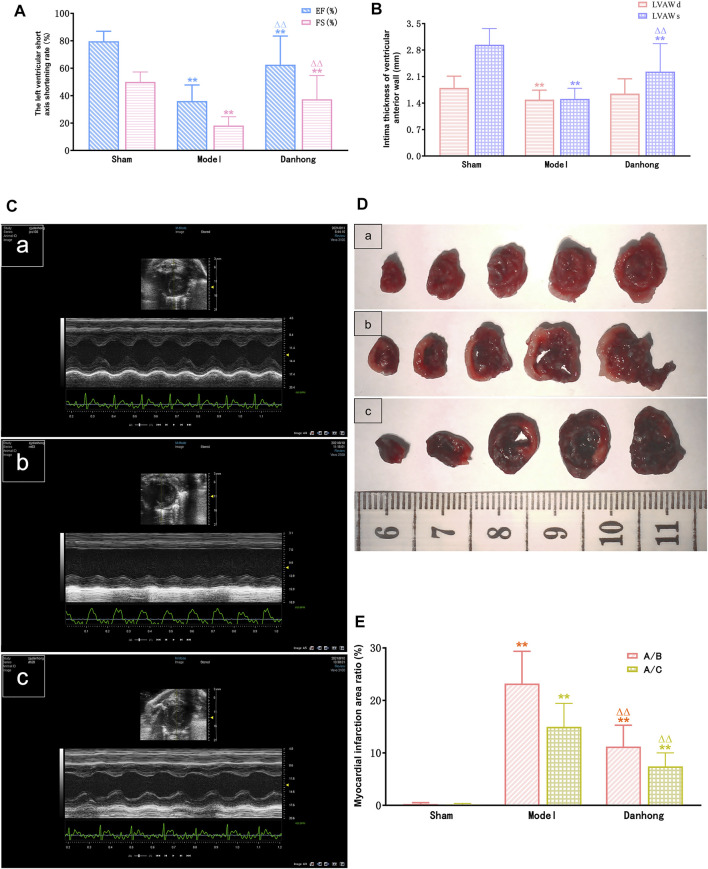
**(A)** The effect of DHI on ejection fraction and left ventricular short axis shortening rate in AMI model rats ( $\bar{x}$ ±s). **p < 0.01 vs. sham; ^△△^ *p* < 0.01 vs. model. **(B)** The effect of DHI on end-diastolic and end-systolic ventricular intimal thickness of AMI model rats ( $\bar{x}$ ±s). **p < 0.01 vs. sham; ^△△^ *p* < 0.01 vs. model. **(C)** The effect of DHI on cardiac function in rats with AMI. a: sham group; b: model group; c: DHI 6 mg/kg group. **(D)** The effect of DHI on myocardial infarction size in AMI model rats. a: sham group; b: model group; c: DHI 6 mg/kg group. **(E)** The effect of DHI on myocardial infarction size in AMI model rats ( $\bar{x}$ ±s). **p < 0.01 vs. sham; ^△△^ *p* < 0.01 vs. model.

Compared with the sham group, end-systolic and end-diastolic anterior wall thicknesses (LVAWs and LVAWd) of the model group were significantly decreased, whereas ejection fraction (EF) and left ventricular fractional shortening (FS) were significantly declined (*p* < 0.01). In addition, the LVAWs, EF, and FS of the DHI group were significantly increased compared with the sham group (*p* < 0.05 or *p* < 0.01) (Figures 8A–C and Tables 2, 3).

**TABLE 2:** The effect of DHI on ejection fraction and left ventricular short axis shortening rate in acute myocardial infarction model rats ( $\bar{x}$ ±s).

LViD	*n*	EF (%)	FS (%)
Group			
Sham group	17	79.68 ± 7.25	50.00 ± 7.24
Model group	17	36.10 ± 11.70^a^	18.17 ± 6.42^a^
DHI 6 ml/kg group	24	62.54 ± 20.90^a^ ^,^ ^b^	37.35 ± 17.29^a^ ^,^ ^b^

a p < 0.01 vs. sham.

b p < 0.01 vs. model; LViD, left ventricular internal diameter.

**TABLE 3:** The effect of DHI on end-diastolic and end-systolic ventricular intimal thickness of acute myocardial infarction model rats ( $\bar{x}$ ±s).

IT	*n*	LVAW d (mm)	LVAW s (mm)
Group			
Sham group	17	1.80 ± 0.31	2.94 ± 0.43
Model group	17	1.49 ± 0.25^a^	1.51 ± 0.28^a^
DHI 6 ml/kg group	24	1.65 ± 0.39	2.23 ± 0.74^a^ ^,^ ^b^

a p < 0.01 vs. sham.

b p < 0.01 vs. model; IT, intimal thickness.

### The Scope of Myocardial Infarction in Rats

The results showed that the percentage of myocardial infarction area to ventricular area and total heart area was significantly lower in the sham group than in the model group (23.21 ± 6.14% and 14.97 ± 4.46%, *p* < 0.01). In addition, the percentage of myocardial infarction size to ventricular size and total heart size was significantly lower in the DHI 6 mg/kg group than in the model group (11.21 ± 4.06% and 7.43 ± 2.56%, *p* < 0.01) (Figures 8D,E and Table 4).

**TABLE 4:** The effect of DHI on myocardial infarction size in acute myocardial infarction model rats ( $\bar{x}$ ±s).

Group	*n*	A/B (%)	A/C (%)
Sham group	11	0.26 ± 0.26	0.17 ± 0.18
Model group	12	23.21 ± 6.14^a^	14.97 ± 4.46^a^
DHI 6 ml/kg group	20	11.21 ± 4.06^a^ ^,^ ^b^	7.43 ± 2.56^a^ ^,^ ^b^

A, myocardial infarction size; B, ventricular size; C, total heart size.

a p < 0.01 vs. sham.

b p < 0.01 vs. model.

### HE Staining and Masson Cardiology

**FIGURE 9:**
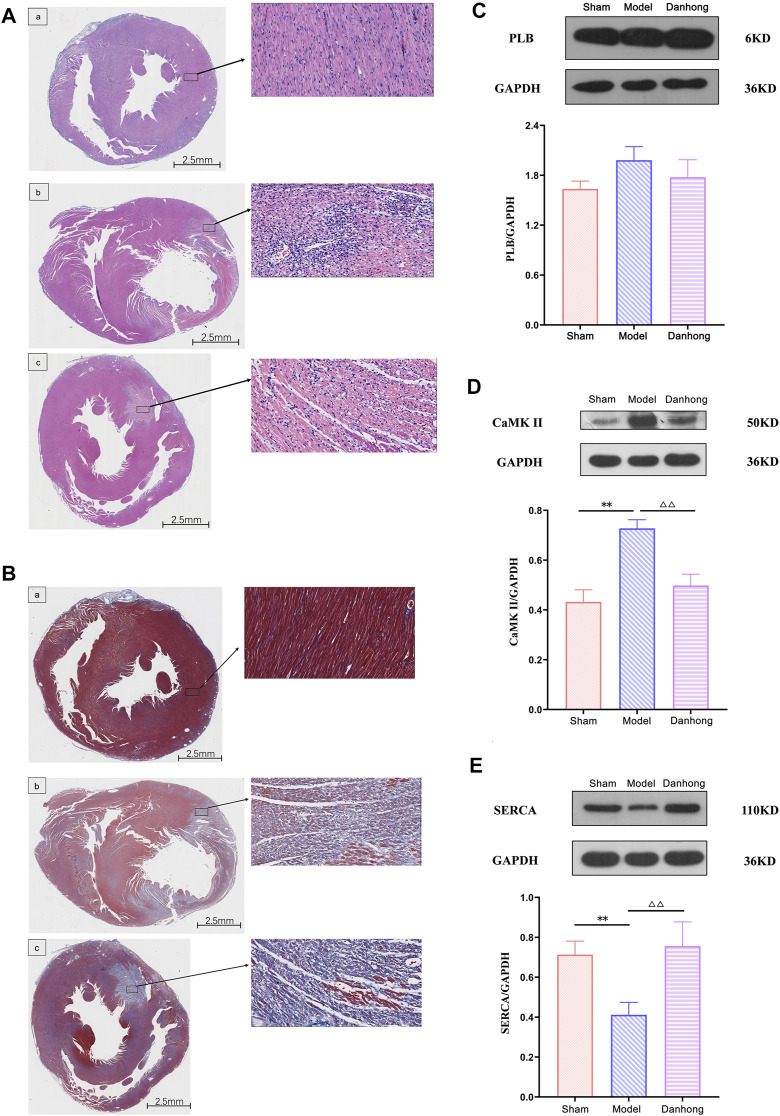
**(A)** The effect of DHI on AMI model rats (HE staining). a: sham group; b: model group; c: DHI 6 mg/kg group. **(B)** The effect of DHI on AMI model rats (Masson staining). a: sham group; b: model group; c: DHI 6 mg/kg group. **(C)** Effect of DHI on the expression of PLB protein in the myocardium of AMI model rats (*n* = 3, $\bar{x}$ ±s). **p < 0.01 vs. sham; ^△△^ *p* < 0.01 vs. model. **(D)** Effect of DHI on the expression of CaMK II protein in the myocardium of rats with AMI (*n* = 4, $\bar{x}$ ±s). **p < 0.01 vs. sham; ^△△^ *p* < 0.01 vs. model. **(E)** Effect of DHI on the expression of SERCA protein in the myocardium of rats with AMI (*n* = 4, $\bar{x}$ ±s). **p < 0.01 vs. sham; ^△△^ *p* < 0.01 vs. model. 14 Table legends.

The results showed that the myocardium in the sham group was intact, the muscle fibers were neatly arranged, and the shape was as usual. There was no degeneration or necrosis. Also, there was no congestion or hemorrhage in the interstitium, no obvious infiltration of inflammatory cells, and no deposition of collagen fibers. However, the pathological findings of the model group showed disordered myocardial fiber arrangement, widened muscle fiber spacing, focal edema and degeneration of myocardial cells, necrosis of individual cells, interstitial vascular proliferation, hyperemia, edema, large number of inflammatory cell infiltration, and extensive deposition of collagen fibers. It is worth noting that the pathological morphology of the myocardial tissue in the DHI 6 mg/kg group showed an orderly arrangement of myocardial fibers, no cell necrosis in the interfibrous septum, a small number of myocardial cells with mild edema and degeneration, a small number of inflammatory cells scattered in the interstitium, and a small area of collagen fiber deposition (Figures 9A,B).

### Protein Blot Assay Protein Expression

The results showed that there was no significant difference between each group in the expression of PLB. Compared with the model group, the expression of CaMK II was significantly downregulated (*p* < 0.01), and the expression of SERCA was significantly upregulated in the DHI group (*p* < 0.01). Compared with the sham group, CaMK II expression was significantly upregulated (*p* < 0.01), and SERCA expression was significantly downregulated in the model group (*p* < 0.01) (Figures 9C–E and Table 5).

**TABLE 5:** Effect of DHI on myocardial protein expression in rats with AMI ( $\bar{x}$ ±s).

Group	PLB (*n* = 3)	CaMK II (*n* = 4)	SERCA (*n* = 4)
Sham group	1.64 ± 0.09	0.43 ± 0.05	0.71 ± 0.07
Model group	1.98 ± 0.16	0.73 ± 0.03^a^	0.41 ± 0.06^a^
DHI 6 ml/kg group	1.78 ± 0.21	0.50 ± 0.05^b^	0.76 ± 0.12^b^

a p < 0.01 vs. sham.

b p < 0.01 vs. model.

## Discussion

In recent years, DHI has received extensive attention in the treatment of AMI (Zou et al., 2018). Pharmacological studies had shown that DHI has a beneficial effect on inhibiting platelet aggregation, improving hemodynamic status, and endothelial function in patients with AMI (Bi et al., 2019). Therefore, this study aims to explore the potential mechanism of DHI in the treatment of AMI.

In this study, compounds integration, potential target prediction, network construction, enrichment analysis, and molecular docking revealed the mechanism of DHI in the treatment of AMI at the molecular level. Through a comprehensive analysis of DHI-key compound-key target-pathway network, we can find that caffeic acid, ferulic acid, rosmarinic acid, salvianolic acid B, danshensu, cytidine and salvianolic acid A are closely related to the ten key targets of MAPK1, MAPK3, PIK3CA, MAPK8, JUN, TNF, EGFR, STAT3, SRC and APP. According to the degree value, ferulic acid, caffeic acid and rosmarinic acid ranked the top three among the seven compounds and were analyzed as key compounds.

As a phenolic compound, ferulic acid can scavenge active oxygen, neutralize free radicals, inhibit enzymes that catalyze free radical formation, and increase the levels of antioxidant enzymes (such as superoxide dismutase and catalase) (Xu et al., 2012; Roy et al., 2013; Pérez-Torres et al., 2021). In addition, studies have shown that ferulic acid can reduce the level of reactive oxygen species induced by TNF-α, protect cardiomyocytes by enhancing autophagy, thereby inhibiting cardiomyocyte apoptosis (Li et al., 2020). In the AMI animal model experiment, Huang et al., found that ferulic acid can protect myocardium from ischemic damage by increasing myocardial blood flow in rats, which provides the possibility to discover potential cardioprotective agents (Huang et al., 2009).

Caffeic acid was a kind of hydroxycinnamic acid, which belongs to the polyphenol family, and has a vasodilator effect on coronary arteries (Taubert et al., 2002). Studies had shown that caffeic acid and its phenethyl ester had a wide range of biological activities, with characteristics of anti-oxidation, anti-inflammatory, immunoregulatory, free radical scavenging and anti-angiogenesis (Silva and Lopes, 2020). In addition, when caffeic acid inhibits the oxidation of low-density lipoproteins, it can prevent atherosclerosis. Hishikawa et al., found that caffeic acid phenethyl ester can inhibit NF-κB, TNF-α, and interleukin-2 (IL-2) related genes in the aorta and ameliorate the progression of atherosclerosis in mice lacking apolipoprotein E (Hishikawa et al., 2005).

As one of the most common caffeic acid esters in rosemary, rosmarinic acid has numerous biological activities, such as active oxygen scavenging, anti-oxidation, anti-inflammatory, neuroprotection, and anti-angiogenesis (Elufioye and Habtemariam, 2019). Javidanpour et al., had shown that rosmarinic acid can prevent myocardial hypertrophy, arrhythmia and cardiac dysfunction, after AMI in mice by inhibiting lipid peroxidation and overexpression of Na^+^/Ca^2+^ exchange protein 1 (Javidanpour et al., 2018). Domitrović et al., had shown that rosmarinic acid can inhibit inflammation by inhibiting the NF-κB pathway and down-regulating TNF-α and cyclooxygenase 2 (COX-2) (Domitrović et al., 2013). At the same time, in this study, molecular docking of rosmarinic acid with TNF was performed, and the binding score of this compound was higher, which indicates a better binding affinity.

Mitogen-activated protein kinase (MAPK) can regulate the recruitment of gene transcription, protein biosynthesis, cell cycle progression, apoptosis, inflammation, and other biological functions (Kyriakis and Avruch, 2012). M embers of the MAPK family include MAPK8 (JNK), MAPK14 (p38), MAPK3 (ERK1), and MAPK1 (ERK2). Zhang et al., showed that long-chain non-coding nuclei rich in transcript 1 (lncRNA NEAT1) might regulate the progression of coronary atherosclerotic heart disease through miR-140-3p/MAPK1 (H. Zhang Hui et al., 2020). The MAPK8 (JNK) signaling cascade can activate inflammation and apoptosis, while the ERK (MAPK3/MAPK1) signaling pathway can mediate cell survival and growth (Sun et al., 2015). The activated ERK MAPKcan inhibit cell apoptosis and protect myocardium (Dhingra et al., 2007). At the same time, an animal experiment has shown that ERK exerts a protective effect on the myocardium and plays a vital role in ischemia/reoxygenation-induced cardiac cell apoptosis (Yue et al., 2000).

The PIK3CA gene encodes the catalytic subunit of phosphor inosine 3-kinase 1A (p110-alpha), which plays an important role in the signaling cascade of cell growth, survival, and proliferation (Vanhaesebroeck and Alessi, 2000). Studies have shown that the activity of p110-alpha plays a vital role in angiogenesis. When activated, it can protect the heart from heart failure caused by myocardial infarction, and when lost, it weakens the contractile function of myocardial cells (Graupera et al., 2008; Lu et al., 2009; Lin et al., 2010). Baulina et al., showed that PIK3CA, which is regulated by miR-375, is associated with the pathological process of AMI (Baulina et al., 2018). Zhu et al., showed that JUN is overexpressed in AMI patients, so it is speculated that inhibiting the expression of JUN may contribute to the treatment of AMI (Zhu et al., 2018).

TNF-α was a pro-inflammatory cytokine whose levels are significantly increased in the serum of patients with AMI. It can also activate other cytokines such as IL-1 and IL-6 and coordinate the host tissue response to acute injury (Mahmoud et al., 2019). In addition, Fahim et al., speculated that TNF-α might be an important indicator of the severity of AMI and the occurrence of heart failure (Fahim et al., 2004). According to reports, the number of endothelial progenitor cells (EPC) was negatively correlated with the progression of coronary heart disease (D'Alessandra et al., 2013). EGFR was a kind of EPC mitogen, and its decreased expression leads to a decrease in the number and activity of EPC in the patient’s systemic circulation (Vickers and Noore, 2013; Sun et al., 2014). Wang et al., showed that miR-23a could inhibit EPC activity in patients with coronary artery disease by targeting EGFR (Wang et al., 2016). It is worth noting that EGFR was enriched in the calcium signaling pathway.

The transcription factor STAT3 was a key regulator of cardiac cell communication. It not only had a protective effect on acute myocardial injury but also resisted cardiac remodeling in subacute myocardial infarction and prevented the development of heart failure (Obana et al., 2010; Haghikia et al., 2014; Enomoto et al., 2015). In experimental animal models, STAT3-deficient mice were more sensitive to myocardial ischemia/reperfusion injury, the infarct area of the heart increased and the function and survival of the heart decreased (Hilfiker-Kleiner et al., 2004). As a non-receptor tyrosine kinase, SRC was up-regulated in the heart 7 days after ischemia-reperfusion and is involved in various biological processes such as cell adhesion, cell cycle, and cell migration (Ziehr et al., 2004; Chetty et al., 2015). Some researchers believe that SRC might be a potential target for heart remodeling and heart failure (Pandey et al., 2018). APP has three spliced mRNA subtypes such as APP695, APP751 and APP770. Among them, APP770 was increased in the serum of ACS patients, and the plasma sAPP770 level of AMI patients was significantly higher (Kitazume et al., 2012). Therefore, this study speculated that DHI might have a therapeutic effect on AMI by regulating the expression of MAPK1, MAPK3, PIK3CA, MAPK8, JUN, TNF, EGFR, STAT3, SRC and APP.

The influx of Ca^2+^ from the environment or release from internal stores leads to a very rapid and dramatic increase in cytoplasmic calcium concentration, which was widely used for signal transduction. Jennings first discovered the existence of calcium overload in acute ischemic myocardial injury in 1981, and the significant change in Ca^2+^ concentration was regulated by protein channels (Wu et al., 2021). Studies had shown that salvianolic acid such as danshensu and salvianolic acid B had a protective effect on the heart of AMI, which was closely related to the inhibition of intracellular calcium pathways (Chen et al., 2011; Wang et al., 2011; Zhao et al., 2012; Lin et al., 2016). In a cross-sectional study, Zhang et al., found that serum calcium concentrations were significantly lower in patients with first AMI compared with patients without AMI, further emphasizing the value of serum calcium level in clinical practice (Zhang et al., 2020). Similarly, integrated bioinformatics studies revealed that the calcium signaling pathway plays an important role in AMI (Guo et al., 2021a). In the present study, the enrichment analysis of KEGG pathway showed that the AMI-related targets regulated by DHI were mainly enriched in the calcium signaling pathway and other pathways.

Previous studies had confirmed that phospholamban (PLB), calcium/calmodulin dependent protein kinase II gamma (CaMK II) and sarcoplasmic reticulum Ca^2+^ ATPase (SERCA) play important roles in the calcium signaling pathway in the treatment of AMI (Mourouzis et al., 2013; Streng et al., 2013; Javidanpour et al., 2017). PLB encoded protein was a key regulator of cardiac diastolic function (Te Rijdt et al., 2019). The product of CaMK II was one of the four subunits of an enzyme belonging to the serine/threonine protein kinase family and the Ca^2+^/calmodulin-dependent protein kinase subfamily (Johnson and Hudmon, 2017). Inhibition of CaMK II expression and activation could improve the sudden myocardial infarction pathological processes and reduce cell necroptosis (Zhang et al., 2016; Yang et al., 2018). In the present study, infiltration of inflammatory cells was seen in the myocardial tissue of the model group. It was also found that the expression of CaMK II protein was significantly higher than sham group. After DHI treatment, the expression of CaMK II protein decreased compared with the model group. It indicated that DHI might treat AMI by inhibiting the expression of CaMK II. SERCA plays a key role in cardiomyocyte Ca^2+^ circulation and heart beating. In heart failuret, SERCA is dysfunctional in cardiomyocytes and the ability to re-uptake Ca^2+^ decreases, affecting Ca^2+^ concentration and homeostasis in cardiomyocytes, and protein expression level is significantly reduced (Khayrullina et al., 2020). Similarly, this study showed that the expression of SERCA protein was significantly lower than sham group. After DHI treatment, the expression of SERCA protein increased compared with the model group. These observations also provide evidences that coincided with the previous studies and reinforced the credibility of our results.

These changes in the expression, suggesting that CaMK II and SERCA are involved in the progression of AMI. Additionally, the infiltration of inflammatory cells, disordered cell arrangement, and myocardial cell edema and degeneration in myocardial tissue were improved compared with the model group. This confirms that DHI has a better therapeutic effect on AMI and that its mechanism of action may be related to the calcium signaling pathway, which is also consistent with the systems pharmacology results described above.

In this study, we not only predicted the potential mechanism of DHI in the treatment of AMI by systems pharmacology, but also constructed an animal model to carry out relevant validation experiments. However, this study still had limitations. As some of the data were derived from multiple public databases, more work need to further characterize the function of calcium signaling pathways during DHI treatment of AMI.

## Conclusion

In this study, the potential mechanism of DHI in the treatment of AMI was systematically revealed by systems pharmacology combined with experimental validation. Among them, UHPLC-LTQ-Orbitrap-MS/MS and related databases were used to screen key components and predict targets, including ferulic acid, caffeic acid and rosmarinic acid. Subsequently, biological function and pathway enrichment analysis was performed to construct a network model to explore the potential mechanism of DHI treatment of AMI from shallow to deep, at multi-level and multi-angles. The results of molecular docking confirmed the results of systems pharmacology. Of importance, we found that PLB, CaMK II, SERCA, etc. play pivotal role in DHI mediated AMI inhibition. The experiments also proved that DHI is effective in treating AMI and can reduce the pathological damage of myocardial tissue.

## Data Availability

The original contributions presented in the study are included in the article/Supplementary Material, further inquiries can be directed to the corresponding authors.

## Abbreviations

AMI, Acute myocardial infarction; BP, Biological process; CaMK II, Calcium/calmodulin dependent protein kinase II gamma; CC, Cell component; COX-2, Cyclooxygenase 2; DHI, Danhong injection; GO, Gene Ontology; IL-2, Interleukin-2; KEGG, Kyoto encyclopedia of genes and genomes; MAPK, Mitogen-activated protein kinase; MF, Molecular function; PLB, Phospholamban; SERCA, Sarcoplasmic reticulum Ca^2+^ ATPase.
