# Supplementary material for: Revealing Calcium Signaling Pathway as Novel Mechanism of Danhong Injection for Treating Acute Myocardial Infarction by Systems Pharmacology and Experiment Validation
Source: Front Pharmacol. 2022 Feb 23;13:839936. doi: 10.3389/fphar.2022.839936 (PMC8905633; doi:10.3389/fphar.2022.839936)
Supplement: Supplementary file 1 [file DataSheet1.docx]

Supplementary Table S1: Basic information of 12 main compounds in DHI.

| No. | Compound | Molecular Formula | Degree Value | Structure |
| --- | --- | --- | --- | --- |
| 1 | Salvianolic acid B | C_36_H_30_O_16_ | 27 | 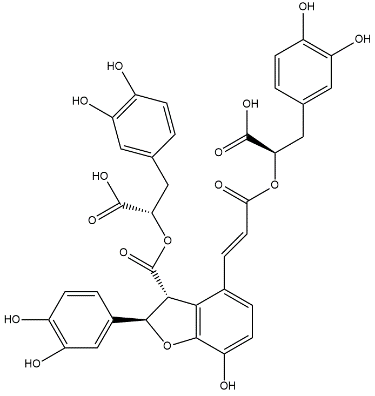 |
| 2 | Salvianolic acid A | C_26_H_21_O_10_ | 59 | 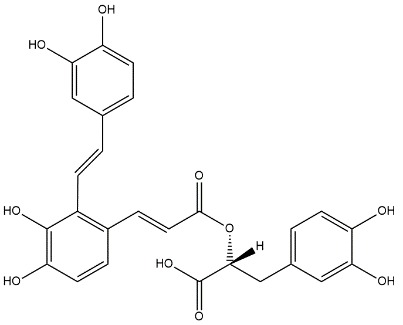 |
| 3 | Rosmarinic acid | C_18_H_15_O_8_ | 57 | 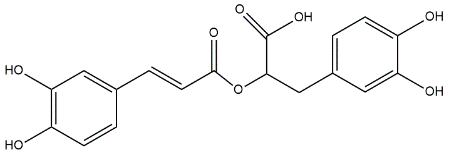 |
| 4 | Danshensu | C_9_H_9_O_5_ | 41 | 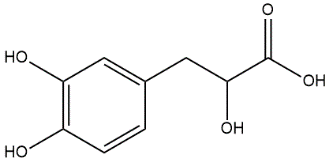 |
| 5 | Uridine | C_9_H_11_N_2_O_6_ | 34 | 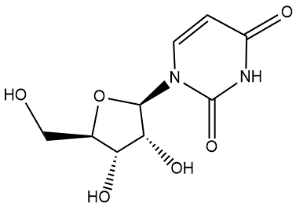 |
| 6 | Hydroxysafflor yellow A | C_27_H_31_O_16_ | 49 | 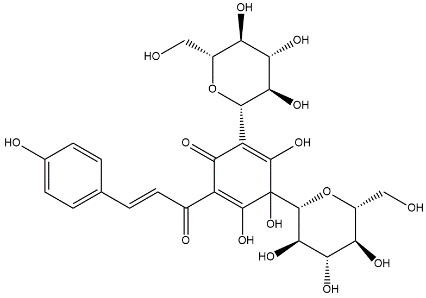 |
| 7 | Caffeic acid | C_9_H_7_O_4_ | 95 | 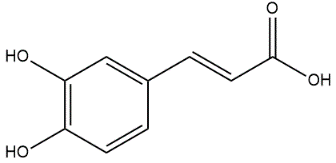 |
| 8 | Protocatechuic acid | C_7_H_5_O_4_ | 53 | 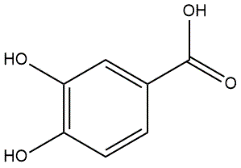 |
| 9 | 3,4-Dihydroxybenzaldehyde | C_7_H_5_O_3_ | 39 | 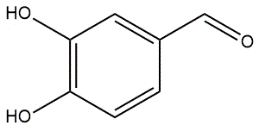 |
| 10 | Cytidine | C_9_H_12_N_3_O_5_ | 47 | 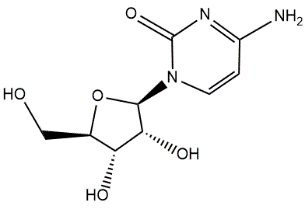 |
| 11 | p-Coumaric acid | C_9_H_7_O_3_ | 40 | 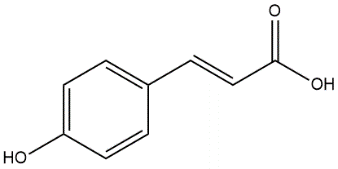 |
| 12 | Ferulic acid | C_10_H_9_O_4_ | 167 | 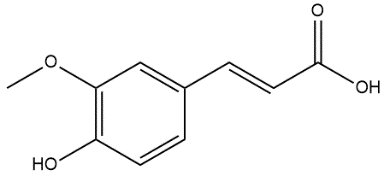 |

Supplementary Table S2: 7 compounds in DHI identified by UHPLC-LTQ-Orbitrap MS/MS

| No. | Compound | Theoretical value (m/z) | MS/MS ions | PPM |
| --- | --- | --- | --- | --- |
| 1 | Salvianolic acid A | 494.1207 | 295.0331,287.2519,313.1674 | -0.148 |
| 2 | Salvianolic acid B | 718.1528 | 519.2232,321.0971,339.1439 | -0.085 |
| 3 | Danshensu | 198.0522 | 179.0041,72.8260 | -0.502 |
| 4 | Protocatechuic acid | 138.0311 | 92.8655,108.9584,119.0216 | -0.000 |
| 5 | Rosmarinic acid | 280.1093 | 160.8447,196.9401,178.8954 | -0.001 |
| 7 | Caffeic acid | 180.0417 | 134.8491,132.9963,150.8149 | -0.000 |

Supplementary Table S3: Basic information of the top ten targets in protein-protein interaction network.

| No. | Target | Name | Betweenness | Closeness | Degree |
| --- | --- | --- | --- | --- | --- |
| 1 | APP | Amyloid-beta precursor protein | 1.17E-01 | 0.5 | 43 |
| 2 | MAPK1 | Mitogen-activated protein kinase 1 | 1.06E-01 | 5.17E-01 | 40 |
| 3 | TNF | Tumor necrosis factor | 7.83E-02 | 5.02E-01 | 40 |
| 4 | MAPK8 | Mitogen-activated protein kinase 8 | 4.77E-02 | 5.12E-01 | 40 |
| 5 | STAT3 | Signal transducer and activator of transcription 3 | 4.30E-02 | 5.03E-01 | 38 |
| 6 | EGFR | Epidermal growth factor receptor | 6.58E-02 | 5.12E-01 | 35 |
| 7 | MAPK3 | Mitogen-activated protein kinase 3 | 5.15E-02 | 5.12E-01 | 35 |
| 8 | PIK3CA | Phosphatidylinositol 4,5-bisphosphate 3-kinase catalytic subunit alpha isoform | 5.01E-02 | 4.65E-01 | 34 |
| 9 | JUN | Transcription factor AP-1 | 2.88E-02 | 4.92E-01 | 33 |
| 10 | SRC | Proto-oncogene tyrosine-protein kinase Src | 2.51E-02 | 4.60E-01 | 33 |
